# Integration of metabolomics and transcriptomics reveals the therapeutic mechanism underlying *Chelidonium majus* L. in the treatment of allergic asthma

**DOI:** 10.1186/s13020-024-00932-y

**Published:** 2024-04-26

**Authors:** Renguang Wang, Xintong Sui, Xin Dong, Liming Hu, Zhimeng Li, Hang Yu, Cuicui Li, Guoxin Ji, Shumin Wang

**Affiliations:** 1 College of Pharmacy, Changchun University of Chinese Medicine, Changchun, 130117 China; 2 Jilin Zhong Ke Bio-Engineering Co., Ltd, Changchun, 130012 China

**Keywords:** *Chelidonium majus* L, Allergic asthma, Metabolomics, Transcriptomics, Energy metabolism, Inflammation

## Abstract

**Background:**

*Chelidonium majus* is a well-known traditional Chinese medicine, and has been reported of the effect in relieving cough and asthma. However, the mechanism of action is still unknown.

**Methods:**

Asthmatic SD rats were first sensitized and established through ovalbumin (OVA) motivation. Subsequently, Hematoxylin and eosin (H&E) staining, Masson’s trichrome (Masson) staining, Periodic acid-Schiff (PAS) staining and inflammatory cytokines assay of interleukin (IL)-4, IL-6, IL-17 were implemented to evaluate the protective effects of *Chelidonium majus* on asthma. Then, the effects of *Chelidonium majus* and their molecular mechanisms of action on asthma were detected based on the integration of transcriptomics and metabolomics analyses.

**Results:**

After administration with *Chelidonium majus*, the histological injuries of inflammation, collagen deposition and mucus secretion in lungs were attenuated and the serum inflammatory cytokines perturbations were also converted. Furthermore, integrated analysis revealed that after *Chelidonium majus* treatment, 7 different expression genes (DEGs) (Alox15, P4ha1, Pla2g16, Pde3a, Nme1, Entpd8 and Adcy9) and 9 metabolic biomarkers (ADP, Xanthosine, Hypoxanthine, Inosine, prostaglandin E2 (PGE2), prostaglandin F2a (PGF2a), phosphatidylserine, Creatine and LysoPC (10:0)) were discovered to be connected with the enrichment metabolic pathways, including Purine metabolism, Arachidonic acid metabolism, Arginine and proline metabolism and Glycerophospholipid metabolism. The obtained metabolic biomarkers and DEGs were mainly related to energy metabolism and inflammation, and may be potential therapeutic targets.

**Conclusion:**

*Chelidonium majus* relieved OVA-induced asthma in rats by regulating the Alox15, P4ha1, Pla2g16, Pde3a, Nme1, Entpd8 and Adcy9 genes expression to restore the disorders in energy metabolism and inflammation.

**Supplementary Information:**

The online version contains supplementary material available at 10.1186/s13020-024-00932-y.

## Introduction

Asthma represents the long-term inflammatory respiratory disorder related to the combination of hereditary and environmental variables [1]. It is commonly triggered by various allergens and mainly manifested as chronic airway inflammation, airway hyperresponsiveness, and airway remodeling [2, 3]. A previous research found that asthma was mainly caused by the imbalanced Th1-to-Th2 cell ratio, which primarily result in T cell differentiation in Th2. Th2 lymphocytes can produce Th2 cytokines, which recruit multiple inflammatory cells, including neutrophils, eosinophils and macrophages to the lesions, and then induce airway inflammation [4–6]. According to the scientific statistics, there were more than 300 million individuals suffering from asthma in the world [7]. Corticosteroids, β-Agonists and antihistamines have been used as main clinical drugs for the treatment of asthma. However, the deficiency of long-term effects and various adverse reactions gradually displayed in the treatment. At the same time, the emergence of drug resistance in patients also limited their use [8, 9]. Therefore, developing drugs with high effectiveness and safety as complementary or alternative medicines is urgent for asthma treatment. Recent studies have suggested that the good therapeutic effects of Chinese herbal remedies for treating asthma have attracted increasing attention [10, 11]. *Chelidonium majus*, a traditional Chinese medicine that spasmolysis and analgesia, relieves coughing and asthma, was first recorded in Salvation Materia Medica and is now in the list of Chinese Pharmacopoeia [12]. In recent years, broadly biological activities of *Chelidonium majus*extracts have been reported, like anti-inflammatory [13], inhibit smooth muscle [14], and anti-bacterial [15] activities. Metabonomics was considered as the “holistic-dynamic-synthesis-analysis” approach, corresponding to dynamic Chinese herbal metabolic processes and holistic concept theory in traditional Chinese medicine [16]. According to reports in literature, a metabolic imbalance was linked to the pathogenesis of asthma [17]. Integrating metabolic and gene expression data is thought to be a novel strategy for displaying the intricate gene and metabolic pathway regulatory networks. For the first time in this research, metabolomics and transcriptomics were incorporated to determine the molecular mechanism of *Chelidonium majus* for asthma.

## Material and methods

### Preparation of the ethanol extract from Chelidonium majus

*Chelidonium majus* (batch number: C19110704) samples were provided by Hebei Renxin Pharmaceutical Co.; Ltd. (Hebei, China), later certified by Prof. Shumin Wang of Changchun University of Chinese Medicine. The *Chelidonium majus* was ground to powder, and put through a filter with a mesh size of 60, after which the precisely weighed powder (400 g) were refluxed twice for a 1-h period with 80% methanol each time. After conflating, filter liquor was subjected to concentration under reduced pressure, freeze-dried into powders, finally storaged at −80 ℃ for future use.

### Ultra-performance liquid chromatography-quadrupole-executive orbitrap mass spectrometry (UPLC-Q-Excutive) analysis of Chelidonium majus extract

Methanol containing 2-Amino-3-(2-chloro-phenyl)-propionic acid (4 ppm) was used to dissolve the extract of *Chelidonium majus*, followed by a filteration with 0.22 μm membrane. And the composition identification was performed by UPLC-Q-Excutive based on literatures [18, 19].

### Animal handling

**Fig. 1:**
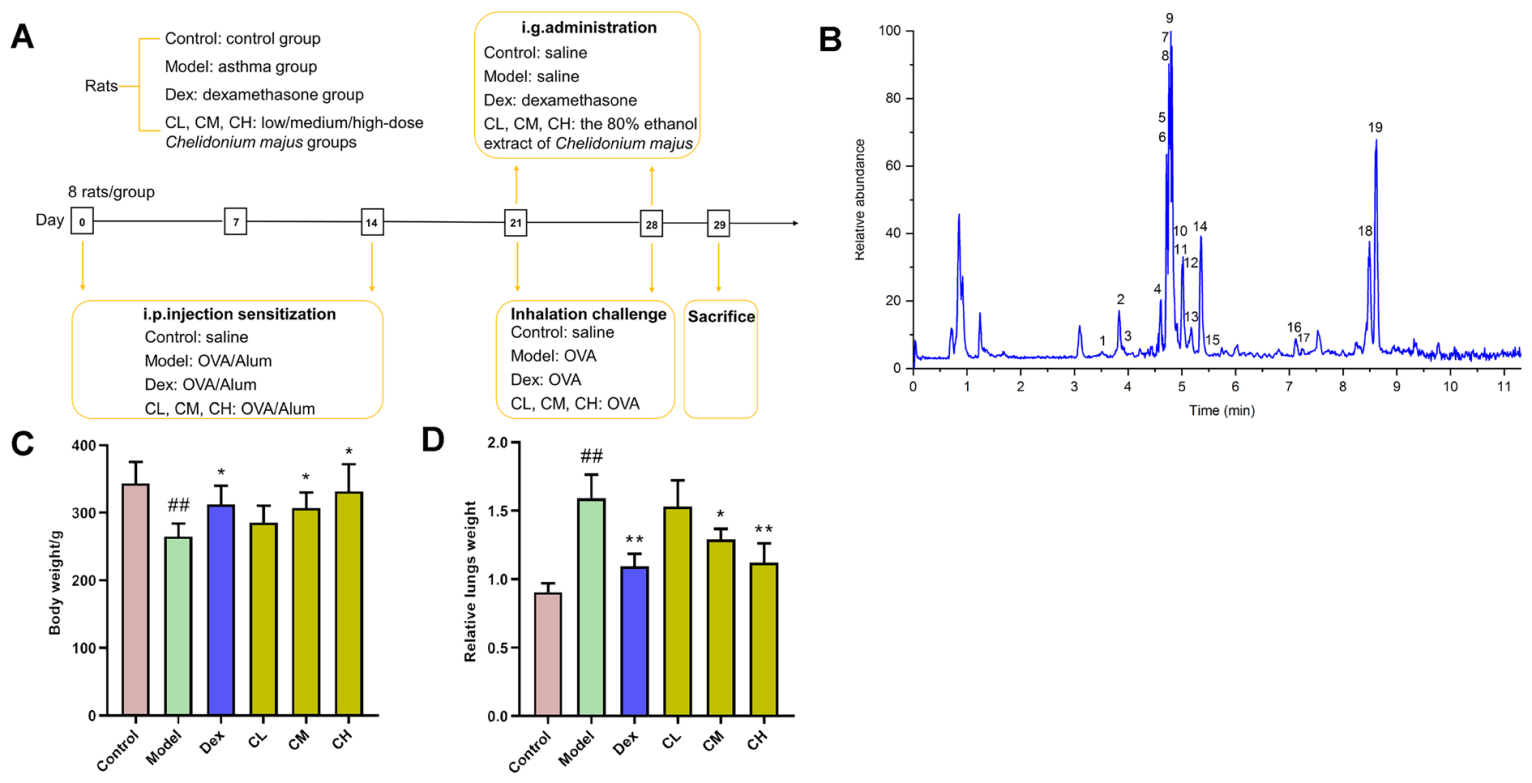
**A** Experimental scheme. **B** Total ion chromatograms ( +) of *Chelidonium majus *extract. **C, D** Body weights and relative lung weights of diverse groups after administration. ##*P* < 0.01 versus Control group; *P < 0.05, **P < 0.01 versus Model group

SD male rats (200 ± 20 g), SPF level, supplied by Changchun Yisi Laboratory Animal Technology Co.; Ltd. [Changchun, China, approval number: SCXK (Ji) 2018–0007]. The rats were kept within the plastic cages under 22 ± 2 ℃, 50 ± 10% humidity, and 12/12-h light/dark cycle conditions. Each animal experiment was carried out following Guidelines for Animal Experimentation of Changchun University of Chinese Medicine (Jilin, China). Our experimental protocols (protocols No. 2020345) gained approval from Animal Ethics Committee of our center. Seventy two rats were randomized as 6 groups (n = 8), namely, control (Control), model (Model), Dexamethasone (Dex), low/medium/high-dose *Chelidonium majus* (CL, CM, CH) groups. Animal model of asthma was established after 7 days of routine feeding. Sensitizing solutions were prepared as follows: 100 mg OVA, 100 mg aluminum hydroxide dissolved into normal saline (1 mL). The rats in each group were intraperitoneal injected of sensitization solution on the 1st and 8th day respectively. Control rats were given injection of 1 mL normal saline in an identical way. According to the pharmacological experimental methodology (4th Edition), the equivalent dose per unit of body weight for rat is 6.3 times that for human. And the daily dose of *Chelidonium majus* for adult human in Chinese Pharmacopoeia is 9 – 18 g [12]. The low, medium, high adult human dose was transformed into the rat dose (9 g × 6.3/70 kg = 0.81 g/kg, 13.5 g × 6.3/70 kg = 1.21 g/kg, 18 g × 6.3/70 kg = 1.62 g/kg). Fifteen days after the last sensitization, each group was continuously gavaged for 7 days, Dex group was administered with dexamethasone (0.5 mg/kg), normal saline was given to Control and Model groups, three different doses of *Chelidonium majus* were given to other groups respectively. The amount of intragastric administration was 10 mL·kg^−1^·d^−1^, and 30 min after each administration, the rats were challenged for a 30 min period with 1% OVA solution (w/v) within the plexiglass chamber once daily. Likewise, normal saline was given to control rats (Fig. 1A). After the last atomization inhalation, each rat was fasted for a 12-h period and anesthetized the next day.

### Pulmonary histopathology

The lung tissues of rats were cleaned with normal saline and fixed with 4% paraformaldehyde. They were then dehydrated in ethylic alcohol, embedded in paraffin, and sliced in 4 μm sections. H&E staining was performed with sections to assess inflammation. Inflammation score was analyzed according to previous description [20, 21]. Masson staining was conducted with sections to evaluate collagen deposition. PAS staining was performed with sections to evaluate goblet cell hyperplasia.

### Serum inflammatory cytokines assay

The serum samples were collected, and the supernatant was subjected to 10 min centrifugation at 3000 rpm. The levels of infammatory factors in serum were evaluated by using ELISA kits, including IL-4 (No. 220805012R, Jiangsu Meimian Industrial Co., Ltd, Yancheng, Jiangsu, China), IL-6 (MM-0190R1, Jiangsu Meimian Industrial Co., Ltd, Yancheng, Jiangsu, China), and IL-17 (No. MM-0088R1, Jiangsu Meimian Industrial Co., Ltd, Yancheng, Jiangsu, China).

### Serum metabolomic profiling

Serum samples were separated chromatographically with the Acquit UPLC HSS T3-C18 column (Waters Corp.; Shanghai, China, 100 mm × 2.1 mm, 1.8 μm) by the Acquit I-Class PLUS instrument (Milford, America). Primary and secondary mass spectrometry data were collected using the Waters Xevo G2-XS QTOF high resolution mass spectrometer in Muse mode by adopting acquisition software (MassLynx V4.2, Waters).

### Data processing, multivariate analysis and statistical analyses

Progenesis QI software was utilized to process raw data to extract and align peaks or implement additional data processing, with mass deviation and theoretical fragment identification being < 100 ppm. Those compounds detected were later searched based on KEGG, lipid maps and HMDB databases to obtain pathway and classification data. In line with grouping, we determined and compared difference multiples, and used T test to determine significant difference *P*-value. OPLS-DA modeling was completed using R language package ropls, then model reliability was tested by 200-fold permutation tests. In addition, multiple cross-validation was used to determine model VIP value. As for OPLS-DA model, its difference multiple, *P*-value and VIP value were combined to screen differential metabolites. Hypergeometric distribution test was used to calculate differential metabolites for the enrichment significance of the KEGG pathway.

### Transcriptomic analysis

This work collected lung tissues from Control (n = 3), Model (n = 3) and CH (n = 3) groups for RNA sequencing. RNA content and quality were analyzed with NanoDrop 2000 (Thermo Fisher Scientific, Wilmington, DE). Besides, Agilent Bioanalyzer 2100 system (Agilent Technologies, CA, USA) was employed for assessing RNA integrity with RNA Nano 6000 Assay Kit. The cDNA library was constructed and evaluated for quality using Agilent Bioanalyzer 2100 system (Agilent Technologies, Inc.).

### RNA sequencing and data analysis

Differential expression was analyzed by DESeq2 R package upon the criteria of *P* < 0.05 and |log2(FoldChange)|> 1. Gene function was annotated based on KO (KEGG Ortholog) databases.

### Immunohistochemistry (IHC) staining

Lung tissue sections were fixed in, dewaxed in dewaxing solution, and dehydrated in an ethanol series. After natural cooling, the sections were placed in PBS (PH7.4) to repair antigen and block endogenous peroxidase. The tissue was uniformly covered with 3%BSA in the tissue chemical circle and closed at room temperature for 30 min. Add PBS to the sections in a certain proportion of Phosphoinositide 3-kinase (PI3K) (Abcam, ab191606, 1:200), Akt (Abcam, ab179463, 1:200), nuclear factor kappa-B (NF-κB) p65 (Abcam, ab16502, 1:500), p-NF-κB p65 (Affinity Biosciences, AF2006, 1:250), IKB-α (Proteintech, 10268-1-AP, 1:250) antibodies, and the sections were placed flat in a wet box at 4 °C for overnight incubation. Corresponding secondary antibodies were added for incubation under ambient temperature for 50 min. After hematoxylin counterstain and ethanol dehydration, the sections were sealed by neutral gum with a cover glass and interpreted under a white light microscope. Intensities were quantified with Image Pro Plus 6.0 software. Three separate fields from each section and three tissue samples from each group were assessed.

### Establishment of the DEGs-based protein–protein interaction (PPI) network and hub gene analyses

PPI network of Model-vs-Control and CH-vs-Model groups overlapping DEG was constructed based on STRING database, followed by importing in Cytascape 3.9.1 platform for network visualization. In addition, CytoNCA plug-in was adopted for counting node topological parameters within PPI network for further hub gene screening.

### Quantitative real-time PCR (qRT-PCR) assays

The hub genes were verified by qRT-PCR. By adopting Trizol reagent, we isolated total tissue RNA in line with commercial protocols. Later, through using SYBR Green Premix Ex TaqII, mRNA levels were detected with β-actin being the amplification reference. The final data were analyzed through 2^−△△Ct^ approach, and primer sequences can be seen from Table 1.

**Table 1:** The primer sequences of hub genes

gene	Former primer	Reverse primer
IL6	GAGTTGTGCAATGGCAATTCTG	ACGGAACTCCAGAAGACCAGAG
IL10	AGAAGCTGAAGACCCTCTGGATA	TTCATTTTGAGTGTCACGTAGGC
H3f3c	TTCAAAACAGACCTGCGCTTC	ATGGATAGCACACAGGTTGGTATCT
CDK1	GAAAGCGAGGAAGAAGGAGTGC	CTGCCAGTTTGATTGTTCCTTTGT
Cxcl2	CTGTACTGGTCCTGCTCCTCCT	AGTGGCTATGACTTCTGTCTGGG
Aurkb	CATCCCTGAGGAGGAAGACCAT	TGCTCCAGAGGCAGTCGTTGT

### Integrating transcriptomics and metabolomics analyses

The present work used MetaboAnalyst 5.0 for integrating transcriptomics and metabolomics data for acquiring a comprehensive relevance between the DEGs and metabolic biomarkers. The qRT-PCR was applied to testify the 10 key genes based on the method expressed before. The sequences of primers were shown in Table 2.

**Table 2:** The primer sequences of key genes

gene	Former primer	Reverse primer
Nme1	ACTACATTGACCTGAAGGACCGC	CCTGTCTTCACAACATTCAGTCCC
Pla2g16	GATATGTGATCCACCTGGCTCC	GTTCAGAGGCAGCGGAGTGTA
Entpd8	TCCCTGAACTACACCCAGAACCT	CATAGAACTGGCCATGCACG
Alox15	TGCTTCTATGCTAAAGACGCCC	GCCAAGATGGACGGAAGAGT
Pde3a	CAGCATAAAGCCACATGAAGCC	ACAGCATAGGACGAAGTGAAGGAC
Rrm2b	TTTCAGTACCTGGTAAACAAGCC	ATGACTGCAAATCGCTGATACTC
Pde7b	GGCTCCTACCCGTTCATTGACT	GTCCAAGGTAGTCTTCGTCCAGC
P4ha1	AGATCCAGAAGGGTTTGTCGG	CAACCTGGTCTTCGTCGTTAGG
Adcy9	TGAGACCTTCGGTTACCATTTCC	GACCTCACCTGAGACATGACAAAC
Ptgs2	CTGATGACTGCCCAACTCCC	CTGGGCAAAGAATGCGAACA

### Statistical analysis

Graphpad prism 8 was employed for analyzing pharmacodynamic profiles. Results were represented by mean ± SEM ($\overline{{\text{x}}}$ ± s). Inter-group differences were evaluated by one-way ANOVA, where *P* < 0.05 and *P* < 0.01 indicated statistical significance.

## Results

### Chemical analysis of Chelidonium majus extract

*Chelidonium majus* extract was analyzed by UPLC-Q-Excutive (Fig. 1B). 19 compounds were identified under positive ion mode, including Magnocurarine, Magnoflorine, Chelamine, Protopine, Allocryptopine, Chelidonine, (S)-N-Methylstylopine, Tetrahydrocoptisine, Coptisine, Homochelidonine, Norchelidonine, Sanguinarine, Berberine, Chelerythrine, Chelilutine, Oxysanguinarine, 6-Methoxydihydrosanguinarine, Dihydrochelerythrine, Corysamine. Additional file 1: Table S1 showed the detailed information with retention time, molecular formula, MS fragment of these compounds.

### Chelidonium majus increased the body weight and decreased the relative lung weight in OVA-induced asthmatic rats.

There was no obvious variation of initial body weight or relative lung weight among the rats of 6 groups. After administration, the body weight and relative lung weight of Control group rats were 343.96 ± 31.39 g and 0.91 ± 0.06. OVA challenge lead to an evident reduction of body weight to 264.85 ± 19.23 g (*P* < 0.01) whereas a remarkable increase of relative lung weight to 1.59 ± 0.17 (*P* < 0.01) in Model group rats. Administration with dexamethasone, medium and high dose of *Chelidonium majus* displayed a remarkable elevation in body weight to 312.53 ± 27.66 g, 307.11 ± 23.11 g, and 331.80 ± 40.38 g (*P* < 0.05) whereas suppression in relative lung weight to 1.09 ± 0.09, 1.29 ± 0.08, and 1.12 ± 0.14 (*P* < 0.01, *P* < 0.05,* P* < 0.01) (Fig. 1C, D). 

### Chelidonium majus alleviated inflammation, collagen deposition and mucus hypersecretion in OVA-induced asthmatic rats

**Fig. 2:**
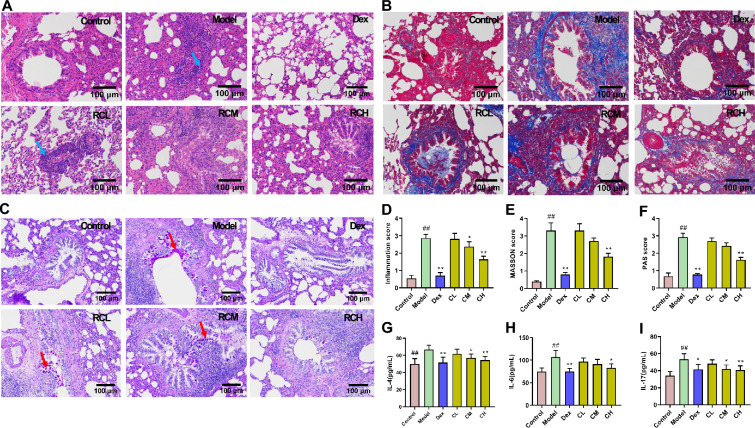
*Chelidonium majus* reduced pulmonary lesions and serum inflammatory cytokines levels. **A** H&E staining (magnifcation × 200), blue arrows indicate inflammatory cell infiltration. **B** Masson staining (magnifcation × 200). **C** PAS staining (magnifcation × 200), red arrows indicate goblet cell proliferation. **D** inflammation score. **E** Masson score. **F** PAS score. **G**–**I** Inflammatory cytokines assay. Data are represented by mean ± SEM (n = 8). ##*P* < 0.01 versus the Control group; *P < 0.05, **P < 0.01 versus the Model group

Control group did not show abnormal findings of the structure in the bronchial and alveolar. Nevertheless, model rats showed an obviously increased thickness in the alveolar septum, and tremendous inflammatory cell infiltrations in bronchi and perivascular. Intragastric administration of Dex, medium and high dose of *Chelidonium majus* all apparently alleviated the severity of pulmonary lesion compared with Model group (*P* < 0.01, *P* < 0.05, *P* < 0.01). (Fig. 2A–D). As revealed by Masson staining, the collagen deposition of Model group was severe. Administration with Dex and high dose of *Chelidonium majus* both markedly alleviated the collagen deposition in OVA-induced rats (*P* < 0.01) (Fig. 2B–E). Based on PAS staining, there was few goblet cells and little mucus secretion in Control group. On the contrary, there were massive goblet cells proliferate on airway epithelium, and the mucus hypersecretion can be seen in the inner wall of airway in Model group. And the goblet cell proliferation and mucus secretion were attenuated in Dex, CH groups rats (*P* < 0.01). (Fig. 2C–F). The results indicated that high dose of *Chelidonium majus* obviously attenuated the pulmonary histopathology of asthmatic rats.

### Chelidonium majus regulated inflammatory cytokines production within OVA-induced asthmatic rats

According to statistical analyses, IL-4, IL-6, and IL-17 levels (66.87 ± 4.93 pg/mL, 106.95 ± 14.41 pg/mL, and 53.35 ± 6.41 pg/mL) were signifcantly higher in Model group relative to Control group (49.78 ± 6.42 pg/mL, 74.57 ± 8.11 pg/mL, and 34.27 ± 4.69 pg/mL). Inversely, in comparison with Model group, IL-4, IL-6, and IL-17 levels reduced obviously to 51.60 ± 6.31 pg/mL, 74.98 ± 6.65 pg/mL, 41.57 ± 5.69 pg/mL in Dex group (*P* < 0.01) and 54.47 ± 4.05 pg/mL, 82.86 ± 8.97 pg/mL, 40.68 ± 4.96 pg/mL in CH group (*P* < 0.01, *P* < 0.05, *P* < 0.01). IL-4 and IL-17 levels reduced obviously to 56.99 ± 4.39 pg/mL and 41.90 ± 4.88 pg/mL in CM group (*P* < 0.05) (Fig. 2G–I). These findings demonstrated that *Chelidonium majus* exhibited good performance in anti-inflammatory effect, and high dose was more effective than medium dose.

### Metabolomic analysis

**Fig. 3:**
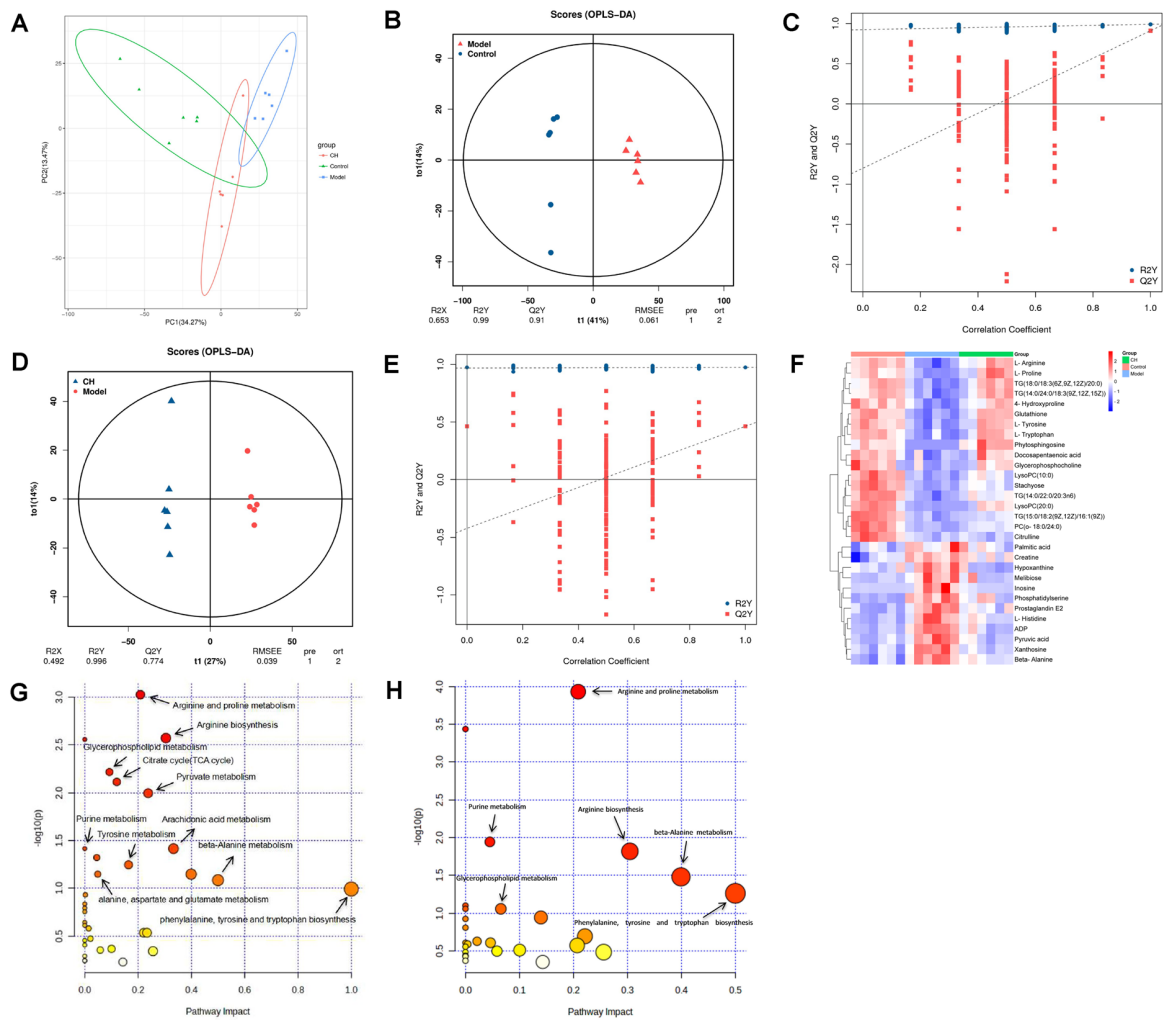
Metabolomic results. **A** PCA of metabolic profiles. **B**–**E** Diferential metabolite analysis model between groups based on OPLS-DA. **F** Heatmap visualization for metabolic biomarkers in Control, Model and CH groups (n = 6). **G, H** Metabolic pathway analysis of groups based on KEGG

Control group was significantly separated from Model group. CH group was basically separated from Model group and approached Control group, based on PCA score plots (Fig. 3A). According to these results, there were significant differences in metabolite profiles of CH group relative to Model group, and CH group manifested a better performance of the recovery from OVA-induced metabolic disturbance. The parameters R2Y, Q2 values of OPLS-DA were approached to 1 (0.99 and 0.91), which implied the presence of less unrelated variables of OPLS-DA models. For checking reliability of OPLS-DA model, replacement tests were carried out. R2 and Q2 values remarkably decreased compared with original points, which was indicative of the asthma model being well built (Fig. 3B–E). According to the multivariate analysis of OPLS-DA with the parameters settings of Fold Change (FC) > 1, variable importance in projection (VIP) > 1 and* P* < 0.05, we detected 46 candidate serum metabolites to be metabolic biomarkers that mainly associated with lipid, organic acid, fatty acid, and amino acid. Compared with Control group, 15 metabolites in Model group showed up-regulated, whereas 31 showed down-regulated. When compared with Model group, 12 metabolites of CH group were down-regulated, 18 metabolites were up-regulated (Additional file 2: Table S2). The metabolite heatmap is shown in Fig. 3F.

MetaboAnalyst 5.0 was used to analyze pathways of all metabolic biomarkers with the filter Criteria of −log P > 1 and Impact > 0. 11 main metabolic pathways were determined in Control-vs-Model group, included Arginine and proline metabolism, Citrate cycle, Pyruvate metabolism, Purine metabolism, Arachidonic acid metabolism, Arginine biosynthesis, Tyrosine metabolism, Sphingolipid metabolism, beta-Alanine metabolism, Phenylalanine, tyrosine and tryptophan biosynthesis, and Alanine, aspartate and glutamate metabolism. 6 major metabolic pathways could be detected from Control-vs-Model and CH-vs-Model groups, included Arginine and proline metabolism, Arginine biosynthesis, Purine metabolism, beta-Alanine metabolism, Glycerophospholipid metabolism, and Phenylalanine, tyrosine and tryptophan biosynthesis (Fig. 3G, H).

To sum up, asthma induction disturbed these metabolic biomarkers as well as the associated metabolic pathways. The alteration was mainly associated with lipid, amino acid, and fatty acids metabolic disorders. Following intervention with high dose of *Chelidonium majus*, the disorder was restored to some extent.

## Transcriptomics analysis

### KEGG enrichment analysis

**Fig. 4:**
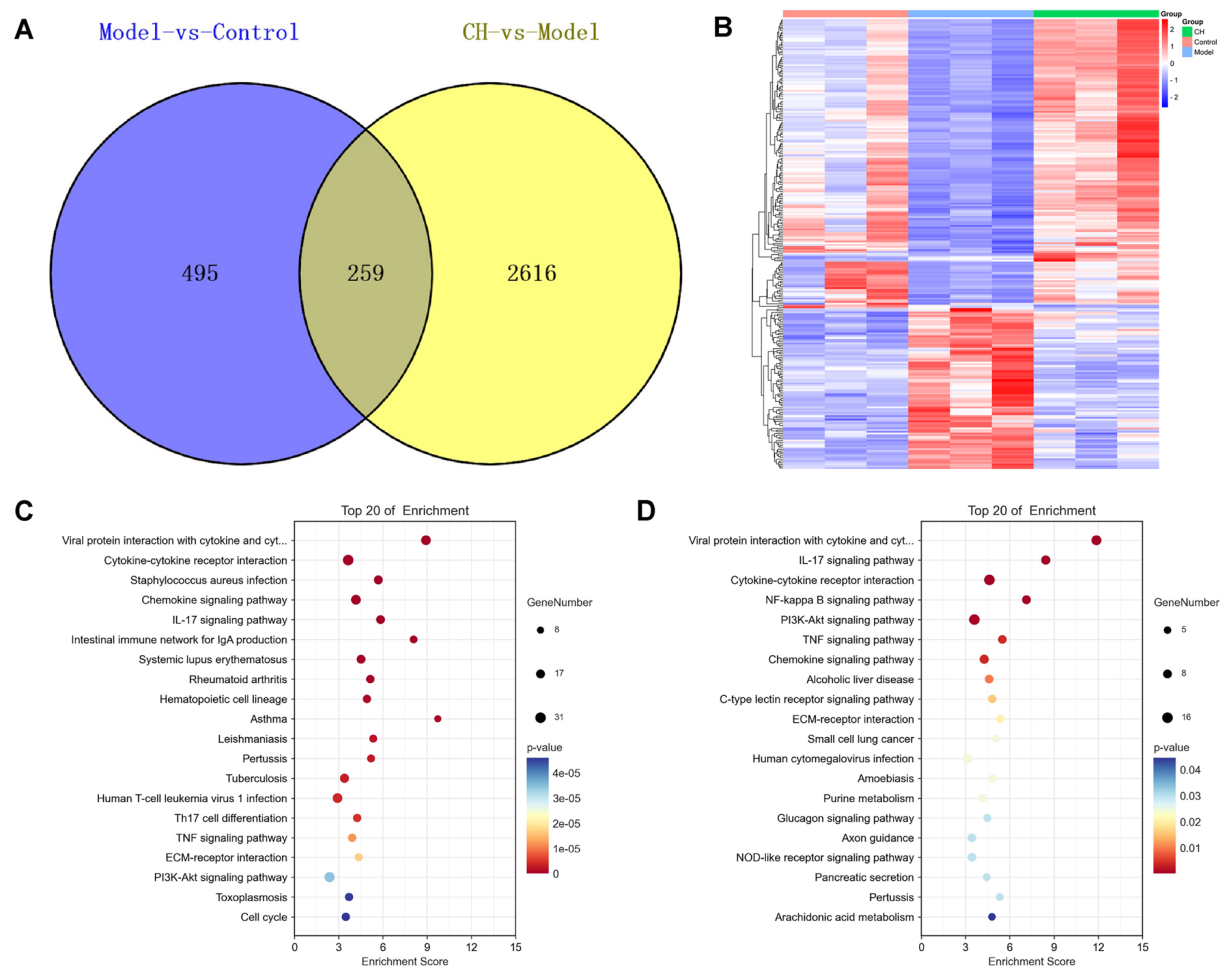
Screening of DEGs and KEGG enrichment (n = 3). **A** Venn diagram showing DEGs in different groups. **B** Heatmap showing DEGs in Control, Model and CH groups. **C, D** KEGG pathway enrichment on DEGs-Bubble chart of Model-vs-Control group and Model-vs-Control and CH-vs-Model groups respectively

There were altogether 754 DEGs detected when comparing Model-vs-Control group, including 454 with up-regulation while 300 with down-regulation. After treatment, there were 260 DEGs regulated in CH group, including 165 showing up-regulation whereas 95 showing down-regulation relative to Model group (Fig. 4A). Heatmap was obtained by clustering the DEGs (Fig. 4B). After high dose of *Chelidonium majus* intervention, gene levels within lung tissue showed a similarity to Control group, indicating the role of *Chelidonium majus* in regulating DEGs levels in Model group. In order to investigate DEGs-regulated biological pathways, we carried out KEGG analysis. Figure 4C, D displayed the 20 most significant pathways. Those pathways enriched were chiefly related to inflammatory pathways (Cytokine-cytokine receptor interaction, Chemokine pathway, IL-17 pathway, TNF pathway, PI3K-Akt pathway, T17 cell differentiation pathway), immune pathways (Intestinal immune network for IgA production pathway, Hematopoietic cell lineage pathway), airway remodeling related signaling pathway (ECM-receptor interaction pathway), energy metabolism pathway (Purine metabolism pathway) and Asthma pathway of Model-vs-Control group. The enrichment pathways were mostly related to inflammation signaling pathways (IL-17 pathway, Cytokine-cytokine receptor interaction, NF-κB pathway, PI3K-Akt pathway, TNF pathway, Chemokine pathway, NOD-like receptor pathway, Arachidonic acid metabolism), energy metabolism pathway (Purine metabolism), airway remodeling related signaling pathway (ECM-receptor interaction pathway) and Pertussis of Model-vs-Control and CH-vs-Model groups.

**Fig. 5:**
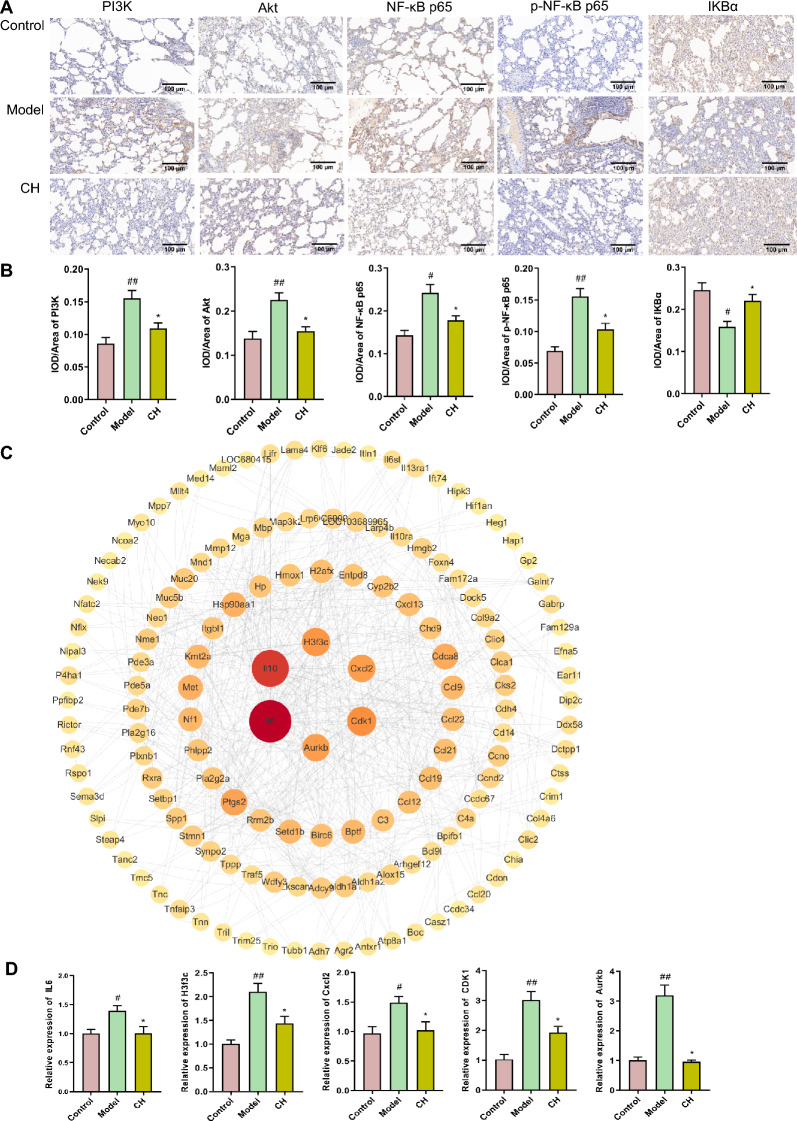
IHC and PPI analysis. **A, B** IHC detection of PI3K, Akt, IKB-α, NF-κB p65, p-NF-κB p65 protein levels within rats lung tissues. The values are represented by mean ± SEM (n = 3). #*P* < 0.05, ##*P* < 0.01 versus Control group; *P < 0.05 versus Model group. **C** PPI networks regarding DEGs in Model-vs-Control and CH-vs-Model groups. **D** hub genes verification. Results are represented by mean ± SEM (n = 3). #*P* < 0.05, ##*P* < 0.01 versus Control group; *P < 0.05, **P < 0.01 versus Model group

We selected PI3K-Akt and NF-κB pathways for verifying the roles of *Chelidonium majus* in resisting inflammation when treating asthma. Compared with Control group, PI3K, Akt, NF-κB p65, p-NF-κB p65 levels had up-regulated significantly (*P* < 0.01, *P* < 0.01, *P* < 0.05, *P* < 0.01), while IKB-α level had down-regulated significantly (*P* < 0.05) in Model group. After high dose of *Chelidonium majus* administration, PI3K, Akt, NF-κB p65, p-NF-κB p65 levels had down-regulated dramatically (*P* < 0.05), IKB-α level had up-regulated evidently (*P* < 0.05) relative to Model group (Fig. 5A, B).

### PPI network establishment and hub gene analyses

Based on these findings, 260 shared DEGs of Model-vs-Control and CH-vs-Model groups were obtained. PPI networks were built through STRING database, followed by visualization with Cytoscape 3.9.1 platform (Fig. 5C). And then according to degree centrality, 6 genes showing the greatest degree values were acquired, including IL-6 (degree = 60), IL-10 (degree = 48), H3f3c (degree = 30), CDK1 (degree = 28), Cxcl2 (degree = 26) and Aurkb (degree = 26) of Model-vs-Control and CH-vs-Model groups.

For validating functions of *Chelidonium majus* in asthma by those potential hub genes, IL-6, IL-10, H3f3c, CDK1, Cxcl2 and Aurkb mRNA expression was detected through qRT-PCR. According to Fig. 5D, OVA-induced asthma caused an obvious upregulation of IL-6, H3f3c, DK1, Cxcl2 and Aurkb (*P* < 0.05,* P* < 0.01, *P* < 0.05, *P* < 0.01, *P* < 0.01) relative to Control group. Administration with high dose of *Chelidonium majus* induced a significant down-regulation of IL-6, CDK1, H3f3c, Cxcl2, Aurkb (*p* < 0.05). Unluckily, IL-10 was not detected.

### Integration of metabolomics and transcriptomics analyses

**Fig. 6:**
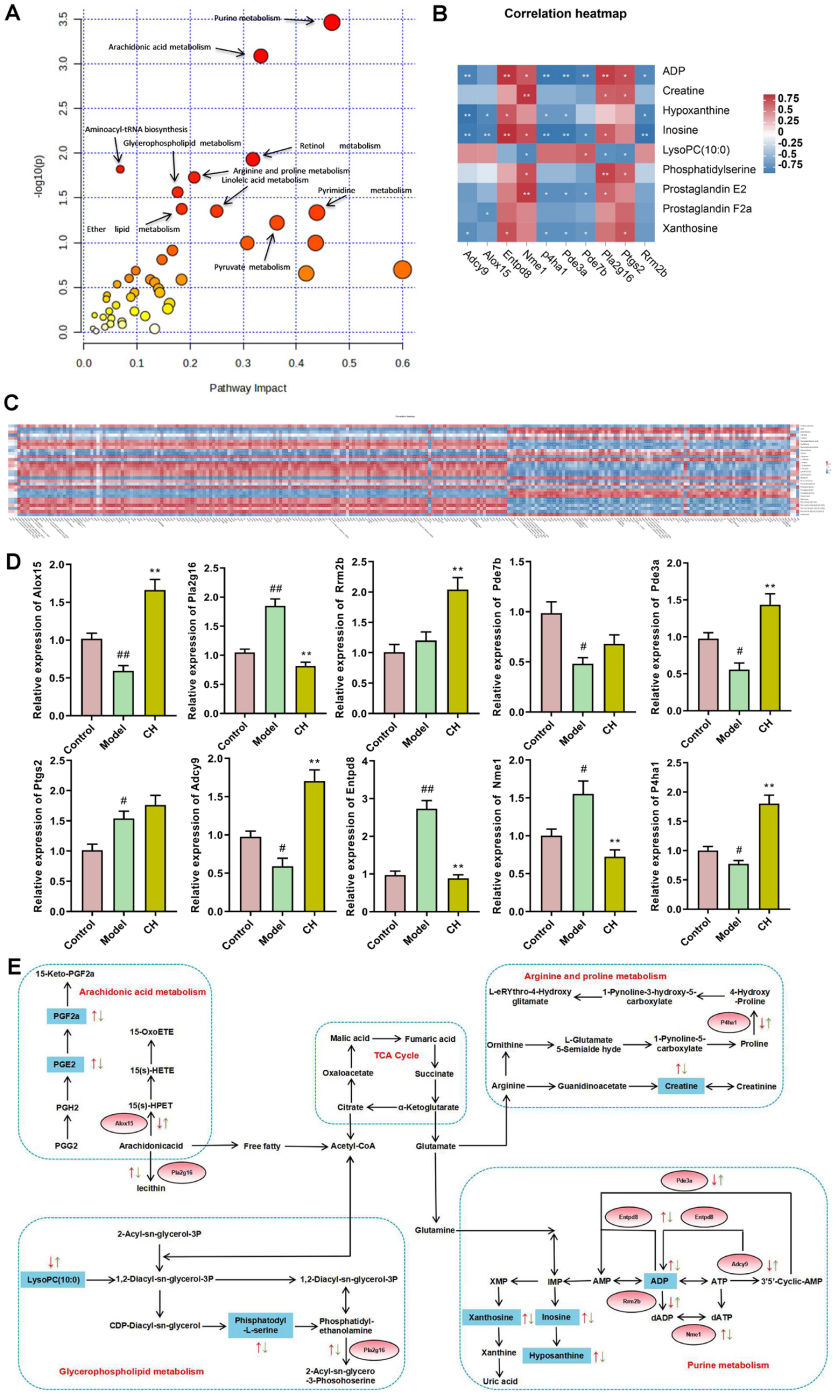
Integration analysis results. **A** Joint-Pathway analysis of Model-vs-Control and CH-vs-Model groups. **B, C** Correlation analysis between metabolic biomarkers and DEGs on the basis of spearman rank. **D** qRT-PCR validation of the 10 genes. Results are represented by mean ± SEM (n = 3). #*P* < 0.05, ##*P* < 0.01 versus Control group; *P < 0.05, **P < 0.01 versus Model group. **E** Changes in metabolites (blue squares) and differential genes (red circles) in the 4 metabolism on the basis of integrated analysis. The red arrow indicates the increase or decrease of Model group relative to Control group. The green arrow reveals the increase or decrease of CH  group relative to Model group

The linked network of metabolic biomarkers and DEGs was displayed in Fig. 6A with the filter Criteria of −log P > 1, mainly included Arginine and proline metabolism, Arginine biosynthesis, Glycerophospholipid metabolism, Citrate cycle (TCA cycle), Pyruvate metabolism, Arachidonic acid metabolism, Purine metabolism, Tyrosine metabolism, Sphingolipid metabolism, beta-Alanine metabolism, and Phenylalanine, tyrosine and tryptophan biosynthesis in Model-vs-Control group and Purine metabolism, Arachidonic acid metabolism, Retinol metabolism, Arginine and proline metabolism, Aminoacyl-tRNA biosynthesis, Glycerophospholipid metabolism, Linoleic acid metabolism, Ether lipid metabolism, Pyrimidine metabolism, and Pyruvate metabolism in both Model-vs-Control and CH-vs-Model groups.

The metabolic biomarkers and DEGs were analyzed using spearman rank correlation (Fig. 6B, C). As was shown, there was noticeable correlation between some metabolites and DEGs. Pde3a, Adcy9 were negatively related with ADP, Inosine (*P* < 0.01), Xanthosine (*P* < 0.05), Hypoxanthine (*P* < 0.05, *P* < 0.01). Pde7b was negatively related with ADP, Inosine, Xanthosine (*P* < 0.01, *P* < 0.05, *P* < 0.05) and Entpd8 was positively related with ADP, Inosine, Xanthosine, Hypoxanthine (*P* < 0.01, *P* < 0.01, *P* < 0.05, *P* < 0.05). Rrm2b was positively related with ADP (*P* < 0.05), Xanthosine (*P* < 0.01), and negatively related with Inosine (*P* < 0.01). Nme1 was positively related with ADP and Inosine (*P* < 0.05). Pla2g16 was positively interrelated with PGE2 and phosphatidylserine (*P* < 0.05, *P* < 0.01) and negatively interrelated with LysoPC(10:0) (*P* < 0.05). Alox15 was negatively interrelated with PGF2a (*P* < 0.05). Ptgs2 was positively interrelated with PGF2a (*P* < 0.05). P4ha1 was negatively associated with Creatine (10:0) (*P* < 0.01). The results supplied a reference for the mechanism of alleviating asthma by *Chelidonium majus*.

Furthermore, to evaluate our results, qRT-PCR was carried out for verifying mRNA levels of those 10 genes (Fig. 6D). As a result, relative to Control group, Ptgs2, Entpd8, Pla2g16 and Nme1 levels within lung tissues in Model group rats had markedly elevated (*P* < 0.05, *P* < 0.01, *P* < 0.01, *P* < 0.05), Adcy9, Alox15, P4ha1, Pde3a and Pde7b levels were notably reduced (*P* < 0.05, *P* < 0.01, *P* < 0.05, *P* < 0.05, *P* < 0.05), but Rrm2b didn’t change. Relative to Model group, administration with high dose of *Chelidonium majus* suppressed Entpd8, Pla2g16 and Nme1 levels within lung tissues (*P* < 0.01), while facilitated Adcy9, Alox15 and P4ha1 levels (*P* < 0.01), but Pde7b and Ptgs2 levels remained unchanged.

The associated DEGs and metabolic biomarkers were displayed in Fig. 6E. 7 DEGs (Alox15, P4ha1, Pla2g16, Pde3a, Nme1, Entpd8 and Adcy9) were discovered related to the enrichment metabolic pathways. P4ha1 was related to Arginine and proline metabolism. Alox15 was associated with Arachidonic acid metabolism, Pla2g16 participated in Glycerophospholipid metabolism, Pde3a, Nme1, Entpd8 and Adcy9 were involved with Purine metabolism.

## Discussion

Cumulative evidence suggested that chronic airway inflammation is the key to the pathogenesis, and is also the basis of airway hyperreactivity and airway remodeling [22]. The inflammatory responses are induced by production of various inflammatory cytokines, such as IL-4, IL6, IL17 that are mediated by Th2 cells [5]. The ELISA assay showed that *Chelidonium majus* could reduce IL-4, IL-6, IL-17 levels in the serum of asthmatic rats. Besides, the alleviation of airway inflammation by *Chelidonium majus* were also observed through H&E analysis, indicating that *Chelidonium majus* may suppress inflammmation through regulating Th2 inflammatory cytokines levels.

Recently, PI3K/Akt/NF-κB signal pathway has been found intimately related to activation and immune responses of T and Blymphocytes, eosinophils and mast cells in the development of asthma [23–25]. As a transcriptional regulator, NF-κB plays an important role in asthma inflammatory pathway. PI3K/Akt can activate the inhibitory protein kinase of NF-κB while accelerating IκB degradation, which contributes to uclear translocation as well as transcriptional activity of NF-κB [26]. IHC analysis suggested *Chelidonium majus* attenuated asthma through restraining PI3K/Akt/NF-κB pathway.

Collagen is a type of extracellular matrix protein, and its deposition on the airway wall signifies the occurrence of airway remodeling [27]. One of the hallmarks of asthma is mucus overproduction from goblet cells [28]. Masson analysis showed that high dose of *Chelidonium majus* inhibited collagen deposition to improve airway remodeling. PAS and the relative weight of lungs analysis demonstrated that high dose of *Chelidonium majus* suppressed the goblet cell proliferation and mucus secretion.

In this study, a prominent metabolic and transcriptional feature observed in asthmatic rats was an alteration to cellular energy metabolism and the expression of related genes in lungs, possibly involved in an increased respiratory burden in providing energy to recruited inflammatory cells. Pde3a, Entpd8, Nme1 and Adcy9 took part in the process of ATP transformation. After administration with high dose of *Chelidonium majus*, the expression of Nme1, Entpd8 and levels of Xanthosine, Hypoxanthine, Inosine, ADP decreased, the expression of Pde3a, Adcy9 increased, indicating that *Chelidonium majus* reversed the disorders of energy metabolism by regulating Purine metabolism.

Besides, various inflammatory cells and inflammatory mediators are active throughout the entire pathogenic course of asthma. Local inflammation is also related with tissue hypoxia due to a integration of reduced and oxygen supply and elevated oxygen requirement of resident and infiltrating cells as a result of the inflammatory response [29]. As was reported that there was a high expression of Alox15 in airway epithelial cells where arachidonic acid was convered to 15-hydroxyeicosatetraenoic acid by Alox15 [30]. PGE2 has been proved to aggravate several inflammatory responses and immune diseases, and PGF2a has been relevant to the acute and chronic inflammation [31–33]. Ptgs2 served as the key enzyme in the biosynthesis of prostaglandin. Phosphatidylserine was investigated to play a vital role in Th2-induced airway hyperreactivity and was closely related with inflammatory cells like eosinophils and macrophages [34]. In this reserch of Model group rats, PGE2, PGF2a, phosphatidylserine levels and Ptgs2 expression elevated, Alox15 level reduced, which possibly aggravated airway inflammation and airway hyperreactivity. Following high dose of *Chelidonium majus* treatment, they all showed obvious reverse levels except for Ptgs2, indicating the attenuation of airway inflammation and airway hyperreactivity. And further investigation needs to be conduct for the unchange of Ptgs2 expression.

Inosine is related to tissue damage, hypoxia and inflammation [35]. Creatine is engaged in energy supply of smooth muscles, and a significant increase of creatine was observed in inflamed lungs [36]. LysoPCs have been reported to be reduced in asthmatic rats and may served as immune suppressors [37]. In this reserch, LysoPC(10:0) displayed reduced level, Creatine and Inosine exhibited increased levels in Model group. The abnormal alteration in Glycerophospholipid metabolism and Arginine and proline metabolism might contributed to inflammation and energy metabolism disorder. After treatment with high dose of *Chelidonium majus*, the abnormal metabolic state was significantly reversed.

## Conclusion

Our current findings demonstrated that *Chelidonium majus* exerted therapeutic effect on airway inflammation, mucus hypersecretion and airway remodeling of OVA-induced asthma. Correlated analysis of transcriptomics and metabolomics studies indicate that 7 DEGs (Alox15, P4ha1, Pla2g16, Pde3a, Nme1, Entpd8 and Adcy9) and 9 metabolic biomarkers (ADP, Xanthosine, Hypoxanthine, Inosine, PGE2, PGF2a, phosphatidylserine, Creatine and LysoPC(10:0)) were involved in the enrichment metabolic pathways of Purine metabolism, Arachidonic acid metabolism, Arginine and proline metabolism and Glycerophospholipid metabolism. And high dose of *Chelidonium majus* may alleviate asthma through impacting on energy metabolism and inflammation. Our study provided a new insight of the therapeutic mechanism for the treatment of asthma by *Chelidonium majus*, which will facilitate the pharmacological research and clinical application of *Chelidonium majus*.

### Supplementary Information

**Additional file 1: Table S1. **UPLC-LC/MS representative information of the 26 compounds under positive ion mode.

**Additional file 2: Table S2. **Identification of metabolic biomarkers in serum.

## Data Availability

The datasets used and/or analyzed during the current study are available from the corresponding author on reasonable request.

## Abbreviations

CH: *Chelidonium majus*
DEGs: Different expression genes
Dex: Dexamethasone
ELISA: Enzyme-Linked Immunosorbent Assay
FC: Fold Change
H&E: Hematoxylin and eosin
KEGG: Kyoto encyclopedia of genes and genomes
IHC: Immunohistochemistry
IL: Interleukin
LysoPC: Lysophosphatidylcholine
Masson: Masson’s trichrome
NF-κB: Nuclear factor kappa-B
OPLS-DA: Supervised orthogonal partial least squares discriminant
OVA: Ovalbumin
PAS: Periodic acid-Schiff
PCA: Principal components analysis
PGE2: Prostaglandin E2
PGF2a: Prostaglandin F2a
PI3K: Phosphoinositide 3-kinase
PPI: Protein–protein interaction
qRT-PCR: Quantitative reverse transcription-polymerase chain reaction
UPLC-Q-Excutive Orbitrap/MS: Ultra-performance liquid chromatography-quadrupole-Executive Orbitrap mass spectrometry
VIP: Variable importance in projection
