# Supplementary material for: Integration of metabolomics and transcriptomics reveals the therapeutic mechanism underlying Chelidonium majus L. in the treatment of allergic asthma
Source: Chin Med. 2024 Apr 26;19:65. doi: 10.1186/s13020-024-00932-y (PMC11055330; doi:10.1186/s13020-024-00932-y)
Supplement: Supplementary file 1 — Additional file 1: Table S1. UPLC-LC/MS representative information of the 26 compounds under positive ion mode. [file 13020_2024_932_MOESM1_ESM.docx]

**Table S1** UPLC-LC/MS representative information of the 26 compounds under positive ion mode

| No. | t_R_/min | Identification | Molecular formular | Experimental mass (MS) | Theoretical mass m/z | Fragment ion (MS/MS) m/z |
| --- | --- | --- | --- | --- | --- | --- |
| 1 | 3.523 | Magnocurarine | C_19_H_24_NO_3_^+^ | 314.1750 | 314.1751 | 269.1169, 175.0754, 143.0490, 107.0495 |
| 2 | 3.837 | Magnoflorine | C_20_H_24_NO_4_^+^ | 342.1699 | 342.1700 | 297.1119, 282.0886, 265.0858, 237,0907, 209.0961, 181.1010 |
| 3 | 3.897 | Chelamine | C_20_H_19_NO_6_ | 370.1284 | 370.1285 | 352.1164, 321.0755, 291.0648 |
| 4 | 4.609 | Protopine | C_20_H_19_NO5 | 354.1332 | 354.1336 | 336.1221, 306.1115, 206.0810, 189.0783, 188.0704, 177.0784, 165.0545, 149.0596 |
| 5 | 4.714 | Allocryptopine | C_21_H_23_NO5 | 370.1645 | 370.1649 | 352.1539, 336.1226, 290.0933, 206.0810, 189.0780, 188.0704, 165.0542, 160.0751 |
| 6 | 4.714 | Chelidonine | C_20_H_19_NO5 | 354.1333 | 354.1336 | 336.1227, 323.0909, 305.0805, 275.0700, 247.0751 |
| 7 | 4.759 | (S)-N-Methylstylopine | C_20_H_20_NO_4_^+^ | 338.1383 | 338.1387 | 190.0861, 188.0705, 149.0599 |
| 8 | 4.759 | Tetrahydrocoptisine | C_19_H_17_NO_4_ | 324.1228 | 324.1230 | 307.0961, 249.0904, 188.0700, 176.0706, 149.0597 |
| 9 | 4.795 | Coptisine | C_19_H_14_NO_4_^+^ | 320.0916 | 320.0917 | 318.0762, 292.0966, 277.0730, 262.0867 |
| 10 | 4.910 | Homochelidonine | C_21_H_23_NO_5_ | 370.1646 | 370.1649 | 352.1537, 290.0934, 275.0703 |
| 11 | 4.910 | Norchelidonine | C_19_H_17_NO_5_ | 340.1181 | 340.1179 | 323.1121, 322.1070, 292.0949 |
| 12 | 5.005 | Sanguinarine | C_20_H_14_NO_4_^+^ | 332.0914 | 332.0917 | 317.0679, 304.0964, 274.0859, 246.0912, 218.0966 |
| 13 | 5.179 | Berberine | C_20_H_18_NO_4_^+^ | 336.1228 | 336.1230 | 321.0989, 320.0914, 306.0758, 292.0964, 278.0803 |
| 14 | 5.351 | Chelerythrine | C_21_H_18_NO_4_^+^ | 348.1228 | 348.1230 | 333.0975, 332.0913, 318.0757, 304.0964, 290.0808 |
| 15 | 5.664 | Chelilutine | C_22_H_20_NO_5_^+^ | 378.1335 | 378.1336 | 363.1097, 362.1010, 348.0862, 332.0904, 320.0906 |
| 16 | 7.121 | Oxysanguinarine | C_20_H_13_NO_5_ | 348.0862 | 348.0866 | 333.0628, 320.0910, 305.0679, 262.0863, 275.0574 |
| 17 | 7.226 | 6-Methoxydihydrosanguinarine | C_21_H_17_NO_5_ | 364.1176 | 364.1179 | 349.0940, 332.0910, 306.0756, 304.0944, 291.0512 |
| 18 | 8.490 | Dihydrochelerythrine | C_21_H_19_NO_4_ | 350.1383 | 350.1387 | 348.1233, 335.1133, 334.1069, 333.1086, 319.1194, 304.0964, 290.0829 |
| 19 | 8.622 | Corysamine | C_20_H_16_NO_4_^+^ | 334.1070 | 334.1074 | 319.0829, 304.0983, 276.1016, 261.0787 |
