# Supplementary material for: Integration of metabolomics and transcriptomics reveals the therapeutic mechanism underlying Chelidonium majus L. in the treatment of allergic asthma
Source: Chin Med. 2024 Apr 26;19:65. doi: 10.1186/s13020-024-00932-y (PMC11055330; doi:10.1186/s13020-024-00932-y)
Supplement: Supplementary file 2 — Additional file 2: Table S2. Identification of metabolic biomarkers in serum. [file 13020_2024_932_MOESM2_ESM.docx]

**Table S2** Identification of metabolic biomarkers in serum

| No. | t_R_/time | m/z | Mass Error(ppm) | Metabolites | Formula | Adduct Ion | VIP | *P* value | Trend | | Pathway (KEGG) |
| --- | --- | --- | --- | --- | --- | --- | --- | --- | --- | --- | --- |
|  |  |  |  |  |  |  |  |  | MvsC | CHvsM |  |
| 1 | 0.59 | 154.0625 | 1.8 | L-Histidine | C6H9N3O2 | [M-H]^-^ | 1.32 | 3.16E-03 | ↓^**^ | ↑^*^ | Histidine metabolism |
| 2 | 0.61 | 124.0082 | 6.7 | Beta-Alanine | C3H7NO2 | [M-H]^-^ | 1.36 | 3.69E-04 | ↑^**^ | ↓^**^ | beta-Alanine metabolism |
| 3 | 0.63 | 176.0565 | 0.7 | 4-Hydroxyproline | C5H9NO3 | [M-H]^-^ | 1.4 | 2.27E-04 | ↑^**^ | ↓^**^ | Arginine and proline metabolism |
| 4 | 0.66 | 132.0779 | 9.1 | Creatine | C4H9N3O2 | [M+H]^+^ | 1.25 | 7.55E-03 | ↓^**^ | ↑^*^ | Arginine and proline metabolism |
| 5 | 0.67 | 116.0717 | 9.6 | L-Proline | C5H9NO2 | [M+H]^+^ | 1.43 | 1.80E-04 | ↑^**^ | ↓^**^ | Arginine and proline metabolism |
| 6 | 0.68 | 346.0448 | -7.1 | Glutathione | C10H17N3O6S | [M+H]^+^ | 1.49 | 2.07E-05 | ↑^**^ | ↓^**^ | -- |
| 7 | 0.68 | 667.2301 | 1.4 | Stachyose | C24H42O21 | [M+H]^+^ | 1.52 | 8.57E-06 | ↑^**^ | ↓^**^ | Galactose metabolism |
| 8 | 0.71 | 426.022 | -0.3 | ADP | C10H15N5O10P2 | [M-H]^-^ | 1.47 | 1.02E-04 | ↓^**^ | ↑^**^ | Purine metabolism |
| 9 | 0.78 | 115.0043 | 5.1 | Fumaric acid | C4H4O4 | [M-H]^-^ | 1.16 | 1.15E-02 | ↓^*^ |  | Citrate cycle (TCA cycle) |
| 10 | 0.78 | 133.0149 | 5.2 | L-Malic acid | C4H6O5 | [M-H]^-^ | 1.32 | 2.18E-03 | ↓^**^ |  | Citrate cycle (TCA cycle) |
| 11 | 0.78 | 87.0099 | 13.1 | Pyruvic acid | C3H4O3 | [M-H]^-^ | 1.32 | 2.19E-03 | ↓^**^ | ↑^**^ | Citrate cycle (TCA cycle) |
| 12 | 0.83 | 427.098 | -5.6 | Phosphatidylserine | C13H24NO10P | [M+H]^+^ | 1.49 | 2.86E-04 | ↓^**^ | ↑^*^ | -- |
| 13 | 1.48 | 535.1524 | -3.5 | Inosine | C10H12N4O5 | [M-H]^-^ | 1.12 | 2.70E-02 | ↓^*^ | ↑^*^ | Purine metabolism |
| 14 | 1.49 | 157.014 | 6.1 | Hypoxanthine | C5H4N4O | [M-H]^-^ | 1.03 | 3.44E-02 | ↓^*^ | ↑^**^ | Purine metabolism |
| 15 | 1.49 | 180.0671 | 2.6 | L-Tyrosine | C9H11NO3 | [M-H]^-^ | 1.47 | 4.93E-05 | ↑^**^ | ↓^*^ | Tyrosine metabolism |
| 16 | 1.76 | 567.1431 | -1.8 | Xanthosine | C10H12N4O6 | [M-H]^-^ | 1.35 | 8.37E-04 | ↓^**^ | ↑^**^ | Purine metabolism |
| 17 | 1.88 | 175.1205 | 8.7 | L-Arginine | C6H14N4O2 | [M-H]^-^ | 1.42 | 3.52E-04 | ↑^**^ | ↓^**^ | Arginine biosynthesis |
| 18 | 1.95 | 495.0879 | -1.9 | CDP-glycerol | C12H21N3O13P2 | [M-H]^-^ | 1.19 | 4.98E-03 | ↑^**^ |  | -- |
| 19 | 2.39 | 205.0985 | 6.6 | L-Tryptophan | C11H12N2O2 | [M+H]^+^ | 1.43 | 2.75E-04 | ↑^**^ | ↓^**^ | Tryptophan metabolism |
| 20 | 2.4 | 381.0737 | -16.7 | Melibiose | C12H22O11 | [M+H]^+^ | 1.29 | 6.68E-03 | ↓^**^ | ↑^*^ | Galactose metabolism |
| 21 | 5.22 | 657.5697 | -8.7 | Phytosphingosine | C18H39NO3 | [M+H]^+^ | 1.53 | 2.30E-04 | ↑^**^ | ↓^**^ | Sphingolipid metabolism |
| 22 | 5.63 | 351.2185 | 2.4 | Prostaglandin E2 | C20H32O5 | [M+H]^+^ | 1.36 | 9.77E-04 | ↓^**^ | ↑^*^ | Arachidonic acid metabolism |
| 23 | 7.05 | 339.2036 | -19.7 | Arachidonic acid | C20H32O2 | [M-H]^-^ | 1.38 | 1.88E-03 | ↓^**^ |  | Arachidonic acid metabolism |
| 24 | 7.5 | 264.2688 | 0.8 | Sphingosine | C18H37NO2 | [M+H]^+^ | 1.38 | 8.64E-04 | ↑^**^ |  | Sphingolipid metabolism |
| 25 | 7.74 | 457.2369 | -18.6 | LysoPC(10:0) | C18H39NO7P+ | [M-H]^-^ | 1.5 | 9.01E-06 | ↑^**^ | ↓^**^ | -- |
| 26 | 7.74 | 596.4016 | 15 | LysoPC(20:0) | C28H58NO7P | [M-H]^-^ | 1.49 | 4.55E-04 | ↑^**^ | ↓^*^ | -- |
| 27 | 7.88 | 255.2334 | 1.8 | Palmitic acid | C16H32O2 | [M-H]^-^ | 1.1 | 1.88E-02 | ↓^*^ |  | Fatty acid biosynthesis |
| 28 | 7.98 | 258.1125 | 9.4 | Glycerophosphocholine | C8H20NO6P | [M+H]^+^ | 1.11 | 1.26E-02 | ↑^*^ | ↓^*^ | -- |
| 29 | 8.17 | 280.2446 | 17.8 | Linoleic acid | C18H32O2 | [M+H]^+^ | 1.11 | 1.24E-02 | ↑^*^ |  | Linoleic acid metabolism |
| 30 | 8.56 | 452.317 | 7.7 | PC(O-14:1(1E)/0:0) | C22H46NO6P | [M+H]^+^ | 1.31 | 1.29E-03 | ↑^*^ | ↑^*^ | -- |
| 31 | 9.37 | 372.2717 | -7.8 | Prostaglandin F2a | C20H34O5 | [M+H]^+^ | 1.18 | 5.07E-03 | ↓^**^ | ↑^*^ | Arachidonic acid metabolism |
| 32 | 9.68 | 532.3755 | -1.3 | LysoPC(20:1(11Z)) | C28H56NO7P | [M+H]^+^ | 1 | 4.27E-02 | ↑^*^ |  | -- |
| 33 | 9.85 | 193.1296 | 0.2 | Citrulline | C6H13N3O3 | [M+H]^+^ | 1.43 | 2.14E-04 | ↑^*^ |  | Arginine biosynthesis |
| 34 | 10 | 259.2075 | 6.8 | Stearidonic acid | C18H28O2 | [M-H]^-^ | 1.31 | 1.05E-03 | ↑^**^ |  | alpha-Linolenic acid metabolism |
| 35 | 10.13 | 611.4244 | 3.3 | Retinol (Vitamin A) | C20H30O | [M+H]^+^ | 1.24 | 1.36E-02 | ↑^*^ |  | -- |
| 36 | 11.32 | 913.7934 | -22 | TG(14:0/22:0/20:3n6) | C59H108O6 | [M+H]^+^ | 1.49 | 8.68E-06 | ↑^**^ | ↓^**^ | -- |
| 37 | 11.32 | 661.4923 | 22.6 | Docosapentaenoic acid | C22H34O2 | [M+H]^+^ | 1.27 | 2.85E-03 | ↑^**^ | ↓^*^ | -- |
| 38 | 11.32 | 856.749 | 12.4 | TG(15:0/18:2(9Z,12Z)/16:1(9Z)) | C52H94O6 | [M+H]^+^ | 1.51 | 7.76E-06 | ↑^**^ | ↓^*^ | -- |
| 39 | 11.33 | 842.7506 | 16.8 | PC(o-18:0/24:0) | C50H102NO7P | [M+H]^+^ | 1.49 | 3.06E-04 | ↑^**^ | ↓^**^ | -- |
| 40 | 11.33 | 870.7591 | 5.4 | TG(16:1(9Z)/16:1(9Z)/20:3(5Z,8Z,11Z)) | C55H96O6 | [M-H]^-^ | 1.18 | 7.07E-03 | ↑^**^ |  | -- |
| 41 | 11.55 | 829.7612 | -13.4 | TG(15:0/16:0/o-18:0) | C52H102O5 | [M-H]^-^ | 1.28 | 1.84E-03 | ↑^**^ |  | -- |
| 42 | 11.62 | 907.7963 | -54.5 | TG(20:0/14:1(9Z)/24:1(15Z)) | C61H114O6 | [M+H]^+^ | 1.3 | 2.54E-03 | ↑^**^ |  | -- |
| 43 | 11.62 | 930.8384 | -11 | TG(14:0/24:0/18:3(9Z,12Z,15Z)) | C59H108O6 | [M+H]^+^ | 1.38 | 3.25E-04 | ↑^**^ | ↓^**^ | -- |
| 44 | 12.01 | 930.837 | -12.5 | TG(18:0/18:3(6Z,9Z,12Z)/20:0) | C59H108O6 | [M+H]^+^ | 1.41 | 1.05E-04 | ↑^**^ | ↓^**^ | -- |
| 45 | 12.16 | 423.3277 | -41.8 | Dodecanoic acid | C12H24O2 | [M+H]^+^ | 1.74 | 2.38E-03 | ↑^**^ |  | Fatty acid biosynthesis |
| 46 | 13.39 | 477.0093 | -70.4 | CDP-Ethanolamine | C11H20N4O13P2 | [M-H]^-^ | 1.28 | 1.46E-03 | ↑^**^ |  | Glycerophospholipid metabolism |
